# Noncanonical Transmission of a Measles Virus Vaccine Strain from Neurons to Astrocytes

**DOI:** 10.1128/mBio.00288-21

**Published:** 2021-03-23

**Authors:** Katrien C. K. Poelaert, Riley M. Williams, Christine M. Matullo, Glenn F. Rall

**Affiliations:** a Fox Chase Cancer Center, Program in Blood Cell Development and Function, Philadelphia, Pennsylvania, USA; b Drexel University College of Medicine, Department of Microbiology and Immunology, Philadelphia, Pennsylvania, USA; Princeton University; Washington University School of Medicine

**Keywords:** ribonucleoprotein, neurotransmitter transporters, astrocyte, measles virus, neuron, synapse

## Abstract

Viruses, including members of the herpes-, entero-, and morbillivirus families, are the most common cause of infectious encephalitis in mammals worldwide. During most instances of acute viral encephalitis, neurons are typically the initial cell type that is infected. However, as replication and spread ensue, other parenchymal cells can become viral targets, especially in chronic infections. Consequently, to ascertain how neurotropic viruses trigger neuropathology, it is crucial to identify which central nervous system (CNS) cell populations are susceptible and permissive throughout the course of infection, and to define how viruses spread between distinct cell types. Using a measles virus (MV) transgenic mouse model that expresses human CD46 (hCD46), the MV vaccine strain receptor, under the control of a neuron-specific enolase promoter (NSE-hCD46^+^ mice), a novel mode of viral spread between neurons and astrocytes was identified. Although hCD46 is required for initial neuronal infection, it is dispensable for heterotypic spread to astrocytes, which instead depends on glutamate transporters and direct neuron-astrocyte contact. Moreover, in the presence of RNase A, astrocyte infection is reduced, suggesting that nonenveloped ribonucleoproteins (RNP) may cross the neuron-astrocyte synaptic cleft. The characterization of this novel mode of intercellular transport offers insights into the unique interaction of neurons and glia and may reveal therapeutic targets to mitigate the life-threatening consequences of measles encephalitis.

## Abstract (precis)

Viruses are the most important cause of infectious encephalitis in mammals worldwide; several thousand people, primarily the very young and the elderly, are impacted annually, and few therapies are reliably successful once neuroinvasion has occurred. To understand how viruses contribute to neuropathology, and to develop tools to prevent or ameliorate such infections, it is crucial to define if and how viruses disseminate among the different cell populations within the highly complex central nervous system.

## INTRODUCTION

The central nervous system (CNS) is the most complex and extensively protected organ system in the mammalian body (1, 2), and yet numerous RNA viruses can gain access to the brain and spinal cord, resulting in debilitating neuropathology. Although neuropathogenesis is rare following infection, the consequences are almost always profound, with lasting health complications which often lead to significant mortality. To understand how viruses, including RNA viruses such as measles, Zika, polio, and influenza, induce neuropathology and/or neurodegeneration, it is crucial to understand how they reproduce and spread among distinct CNS cell types. Many studies have focused on viral transmission between homotypic cells (e.g., neuron-to-neuron), but few have assessed transmission between distinct cell populations, here referred to as heterotypic viral spread. Using measles virus (MV) as a model neurotropic virus, we explored the process of heterotypic spread between neurons and astrocytes.

MV is typically associated with human respiratory tract infection, immunosuppression, fever, myalgia, and a characteristic rash, although in rare cases, MV can infect neurons and glia within the CNS and cause often-fatal neurodegenerative diseases such as subacute sclerosing encephalitis (SSPE). SSPE is a progressive disease that can occur several years after apparent resolution of the acute infection. While we do not know the location or state of the viral genome in the period between acute infection and mortality, some investigators have proposed that MV may establish dormancy within the brain and that SSPE manifests when viral reproduction is reactivated. This hypothesis is consistent with autopsy studies, in which MV infection is associated particularly with neurons, although oligodendrocytes, astrocytes, and infiltrating lymphocytes are also target cells, predominantly at end-stage disease (3–5).

MV is a paramyxovirus of the genus *Morbillivirus*, with a negative-sense, single-stranded RNA genome that encodes six major structural proteins (6). The viral transmembrane glycoproteins, hemagglutinin (H) and fusion (F), are required for viral particle attachment to a target cell receptor and to mediate virus-host cell membrane fusion, respectively (7). These same proteins enable spread between adjacent cells, resulting in formation of giant, multinuclear syncytia.

To date, three MV receptors that are bound by the viral H protein have been identified. Two of these receptors, signaling lymphocyte activation molecule (SLAM) and nectin-4, are used by pathogenic (wild-type) MV strains, whereas human CD46 (hCD46) preferentially enables entry of vaccine strains, such as MV-Edmonston. The cellular distribution of these receptors in humans differs: whereas SLAM is restricted to lymphocytes and nectin-4 is primarily found on epithelial cells, hCD46 is found on most cells, consistent with its role in complement regulation (8–10). However, these receptors are not present in the human brain (11, 12), raising the issue of how MV enters and spreads in cells that do not express known receptors. While it is possible that as-yet-unknown receptors enable entry to neurons and glia, a recent report suggested that MV may spread within cells in a receptor-independent manner. Heterotypic spread of encapsidated measles virus (MV) genomes (ribonucleoproteins [RNPs]) was recently shown (13) between respiratory epithelial cells and primary superior cervical ganglion (SCG) neurons *in vitro*. In that study, epithelial cells expressing nectin-4, but not SLAM, could mediate transfer of RNPs to nectin-1-expressing primary neurons, even though nectin-1 is not a bona fide MV receptor.

Because mice do not bear any known MV receptors, transgenic mice expressing a human viral receptor have been useful in understanding aspects of MV reproduction and pathogenesis. For example, SLAM transgenic mice were used to study the role of type I interferon during wild-type MV infection of glia cells in the CNS (14). Our lab established the first of these transgenic mice, in which the hCD46 vaccine strain receptor was restricted to neurons using a neuron-specific promoter, neuron-specific enolase (NSE) (15). This well-established model mimics the neuropathology observed in humans caused by the wild-type virus (15–19). For example, our recent study showed that MV reproduction in the brain is kept in check by CNS-resident memory T cells. As we show in this report, this state of viral “dormancy,” in which MV replicates to low levels in the absence of overt disease, surprisingly occurs in both neuronal and nonneuronal cells. When the host is immunosuppressed, sentinel immune cells leave the CNS, and virus reproduction and spread resume, resulting in neuropathogenic outcomes (16, 20, 21).

The detection of MV in glia in our mouse model was unexpected, as hCD46 expression is restricted to neurons, and consequently, only neurons are initially susceptible and permissive following intracranial or intranasal challenge with MV vaccine strains (e.g., Edmonston) (22, 23). Defining the mechanism by which MV spreads to otherwise nonpermissive cells was the goal of the experiments presented here.

Astrocytes actively regulate neuronal homeostasis and synaptic plasticity; they can also receive signals from neurons and release neuroactive substances (24, 25). Because of their critical role in regulating neuronal metabolism, fine processes of astrocytes envelop neuronal synaptic terminals, forming a tripartite synapse (26–28). This heterotypic cell interaction enables astrocytes to modulate synaptic neurotransmission (25), for example, via glutamate transporters such as excitatory amino acid transporters (EAATs) that can scavenge excess glutamate within the cleft (29–31).

In this study, we demonstrate that MV RNA and proteins are readily transmitted within cell populations (e.g., neuron-to-neuron; “homotypic”) as well as among cell populations (e.g., neuron-to-astrocyte; “heterotypic”) in a contact-dependent manner. MV transmission between homotypic cell types can occur independently of hCD46 but requires fusion of the donor and recipient cell membranes. In marked contrast, spread between heterotypic cells is fusion independent but glutamate transporter dependent. Moreover, in the presence of RNase A, astrocyte infection is reduced, suggesting that nonenveloped ribonucleoproteins (RNPs) may be present in the neuron-astrocyte synaptic cleft. We hypothesize that expanding the cell populations that can be infected within the CNS may afford the virus long-term shelter during dormancy.

## RESULTS

### MV-infected astrocytes are detected at 30 dpi and are the predominant infected cell type during MV reactivation in NSE-hCD46 mice.

**FIG 1:**
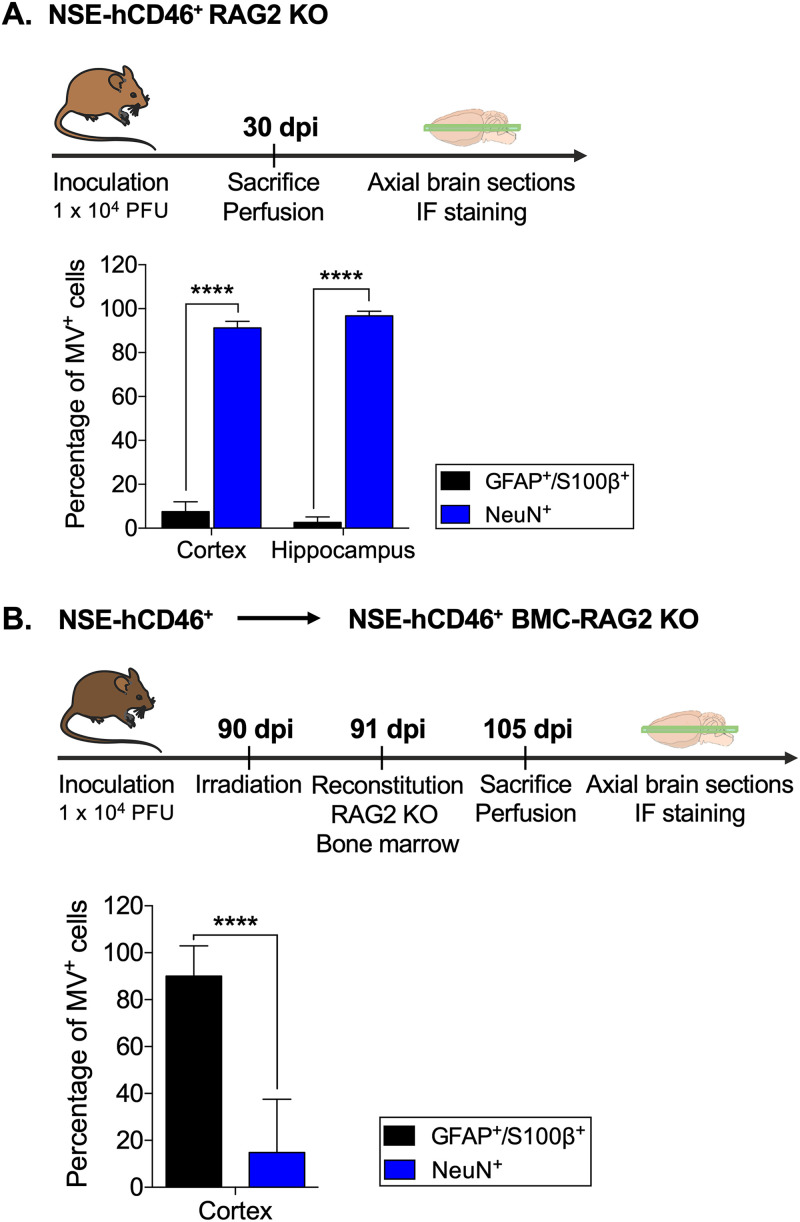
Astrocytes are the predominant infected cell type during MV reactivation in NSE-hCD46 mice *in vivo.* (A) NSE-hCD46^+^/RAG2 KO mice (*n* = 3) were inoculated with 1 × 10^4^ PFU MV-Ed and monitored daily. At 30 dpi, mice were euthanized and perfused with paraformaldehyde, and brains were removed for cryosectioning and immunofluorescence. The number of infected cells within the cortex or hippocampus was then quantified. Results are shown as the number of infected neurons or astrocytes as a percentage of the total number of infected cells seen in 10 brain sections/mouse, with data collected at multiple axial levels. (B) Bone marrow chimeras were generated using NSE-hCD46^+^ recipients (*n* = 2) that had been infected with 1 × 10^4^ PFU MV-Ed at least 90 days previously. Infected mice were reconstituted with RAG2 KO donor bone marrow and monitored daily. At 14 days after transplantation, mice were sacrificed, and brains were treated as described above. The number of infected cells within the cortex was quantified and is shown as the percentage of infected neurons or astrocytes as a proportion of the total number of infected cells, with data derived from 10 brain sections/mouse (*n* = 5). An unpaired *t* test for significance was used. ****, *P* < 0.0001.

In published experiments that are a prelude to these studies, we found that measles virus RNA was detectable in brain tissues of infected NSE-hCD46 transgenic mice months to years after inoculation (16). Moreover, upon immunosuppression, MV reproduction rebounded, suggesting long-term maintenance of replication-competent viral genomes. To assess the location of long-term viral RNA and gene products, we first quantified the cell populations that were infected at various stages of the infection. The upper panels of Fig. 1A and B illustrate the experimental design. For this study, we used 2 mouse genotypes: NSE-hCD46^+^/RAG2 KO (knockout) mice, with an impaired immune response to maximize the CNS infection, and immunocompetent NSE-hCD46^+^ mice. Mice of both genotypes were inoculated intracranially with 1 × 10^4^ PFU MV-Ed and monitored daily. At 30 days postinoculation (dpi), NSE-hCD46^+^/RAG2 KO mice were sacrificed, followed by perfusion, brain sectioning, and immunofluorescence (IF) staining for MV nucleoprotein (MV-N), combined with brain cell markers. In parallel, NSE-hCD46^+^ mice infected for 90 dpi were irradiated lethally and reconstituted with RAG2 KO bone marrow, as previously published (16); this procedure results in MV reactivation from dormancy. At 14 days post-bone marrow reconstitution (105 dpi), mice were euthanized, followed by IF staining of brain sections. During the acute MV infection (30 dpi), MV nucleoprotein (MV-N) was readily detected in the cortex and hippocampus; more than 90% of the infected cells were positive for the neuronal NeuN cell marker. Somewhat unexpectedly, 5 to 10% of the infected cells costained with the astrocyte cell markers (glial fibrillary acidic protein [GFAP] and S100β; Fig. 1A). Reactivated MV was detected in a limited number of cortical cells of the NSE-hCD46^+^/BMC-RAG2 KO mice. Surprisingly, and in contrast to the acute infection, greater than 80% of the infected cells were GFAP/S100β positive, while only 10 to 20% were NeuN positive (Fig. 1B). No other parenchymal cells (e.g., microglia) were positive for viral proteins. These results suggest that astrocytes are the primary harbor for MV during dormancy; presumably, astrocytes are seeded during the primarily neuronal acute infection.

### Primary murine astrocytes lacking hCD46 are not infected directly *in vitro*.

**FIG 2:**
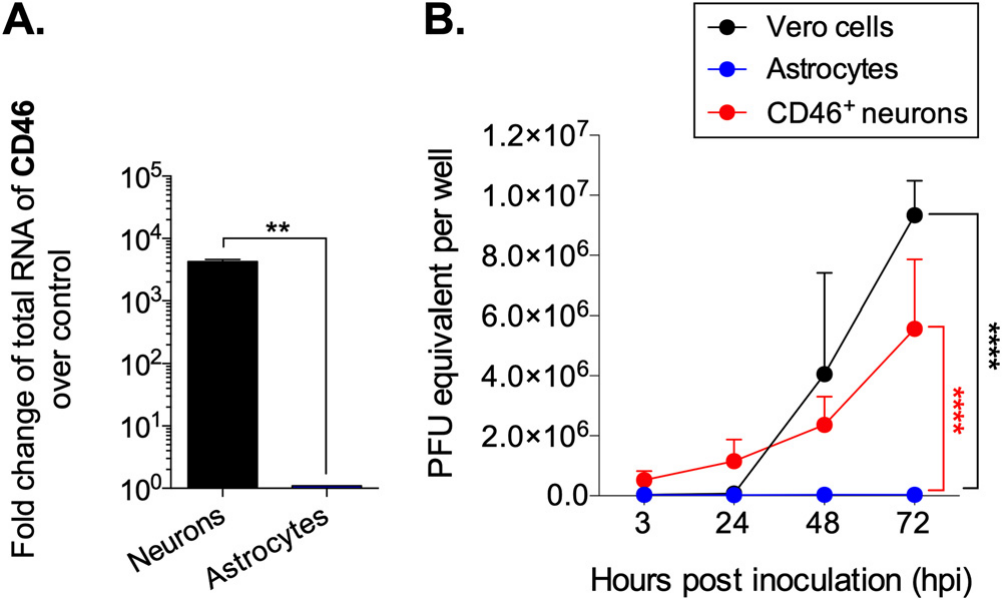
Primary astrocytes lacking hCD46 are not permissive for cell-free MV infection *in vitro*. Primary astrocytes, isolated from NSE-hCD46^+^ mice, were inoculated with MV-Ed (MOI = 1) and collected at 3, 24, 48, and 72 hpi. (A) hCD46 RNA is detected only in neurons from NSE-hCD46^+^ mice; astrocytes do not synthesize hCD46 RNA. Unpaired *t* test for significance was used; **, *P* < 0.01. (B) RNA was collected from inoculated Vero cells, neurons, and astrocytes at the indicated time points. Random hexamers were used as primers for cDNA synthesis, followed by qPCR using primers specific for the MV nucleoprotein (MV-N). Data are represented as PFU equivalent per well (±400,000 cells) based on a standard curve. Data represent means plus SD for three independent experiments. A multiple-way analysis of variance (ANOVA), followed by a Tukey *post hoc* test, was used for significance; ****, *P* < 0.0001.

Given the surprising evidence that astrocytes can become infected with MV in NSE-hCD46^+^ mice in which only neurons bear the viral receptor, we next questioned whether they could be infected directly with cell-free MV particles. Primary murine astrocytes were cultured from NSE-hCD46^+^ embryos and inoculated with MV (multiplicity of infection [MOI] = 1). Vero cells and NSE-hCD46^+^ neurons were included as positive controls; hCD46-negative neurons were used as negative controls. hCD46 is transcribed only in neurons; RNA of hCD46 was not detected in astrocytes (Fig. 2A). Moreover, and in contrast to Vero cells and primary neurons, the RNA levels for MV-N did not increase over time in primary astrocytes (Fig. 2B). We conclude that primary murine astrocytes that lack hCD46 cannot become infected directly with cell-free MV particles. Consequently, the infection of astrocytes observed in our *in vivo* experiments implies that MV infects these cells through a receptor-independent pathway.

### Primary murine astrocytes are susceptible to MV infection upon direct contact with infected hCD46^+^ neurons.

**FIG 3:**
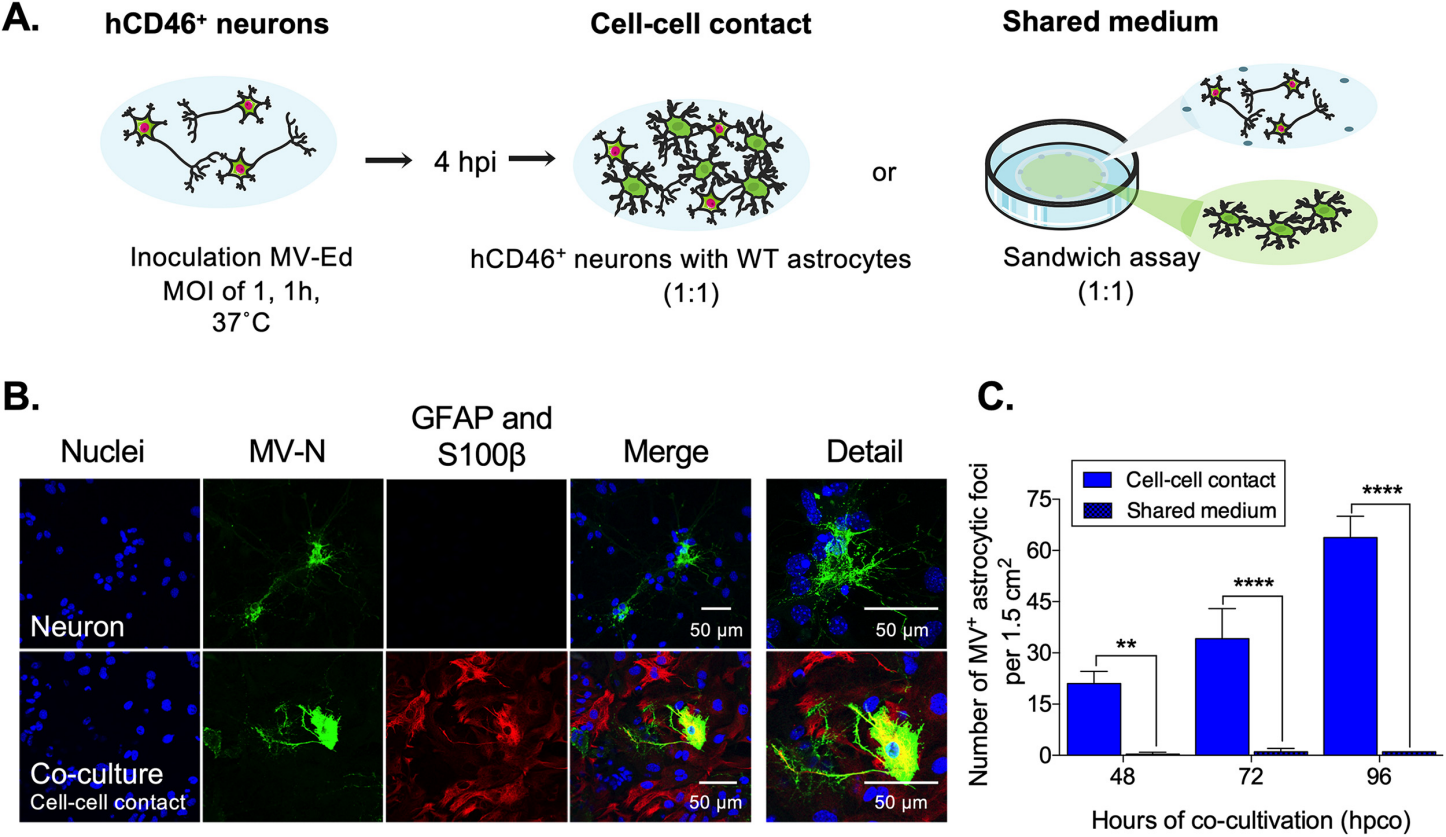
Primary astrocytes are permissive for MV infection when in direct contact with infected hCD46^+^ neurons. Primary neurons were inoculated with MV-Ed (MOI = 1) and cocultured 4 h thereafter either directly (cell-cell contact) or indirectly (shared medium) with uninfected primary astrocytes. (A) Schematic illustration of the experimental setup. (B) At 76 hpi (72 h postcoculture), neuron cultures or neuron-astrocyte cocultures were collected; MV-N and GFAP/S100β astrocyte cell markers were detected by immunostaining with Alexa Fluor 488 (green) and Alexa Fluor 555 (red), respectively; nuclei were counterstained with Hoechst 33342 (blue). (C) The number of astrocytic foci, in both the direct and indirect neuron-astrocyte cocultures, was quantified at the indicated time points. Data represent means plus SD for three independent experiments. An ANOVA, followed by a Tukey *post hoc* test, was used for significance; **, *P* < 0.01; ****, *P* < 0.0001.

To determine how astrocytes become infected, we cocultured primary astrocytes with previously infected neurons (4 h postinfection [hpi]), either in direct contact or indirectly, in shared medium, but otherwise separated without any cell-cell contacts (Fig. 3A). The latter “sandwich” assay was developed because neuron and astrocyte interactions include the exchange of fatty acids and exosomes between the two cell types (32, 33), a mode utilized by other neurotropic viruses to spread within the CNS (34, 35).

At 48, 72, or 96 h postcoinfection (hpco), cocultures were fixed, followed by IF staining for viral proteins and astrocyte cell markers. Pure neuron cultures were included as a positive control and to compare differences in transmission patterns between neurons alone and neuron-astrocyte cocultures. In infected neurons, viral antigen was localized in the soma, axons, and dendrites (Fig. 3B, upper panel). In neuron-astrocyte cocultures, MV-N colocalized with astrocyte markers and was detected throughout the astrocyte cell body (Fig. 3B, lower panel). Significantly more astrocytes became infected when in direct contact with neurons, compared to astrocytes in the sandwich assay (*P* < 0.0001 at 72 and 96 hpco, Fig. 3C).

These data demonstrate that direct neuron-astrocyte contact is required for viral spread from neurons to astrocytes, either through the release and uptake of infectious virus at the cell-cell junction or as an immature viral replication complex.

### The mechanism of MV spread from neurons to astrocytes is cell type specific.

**FIG 4:**
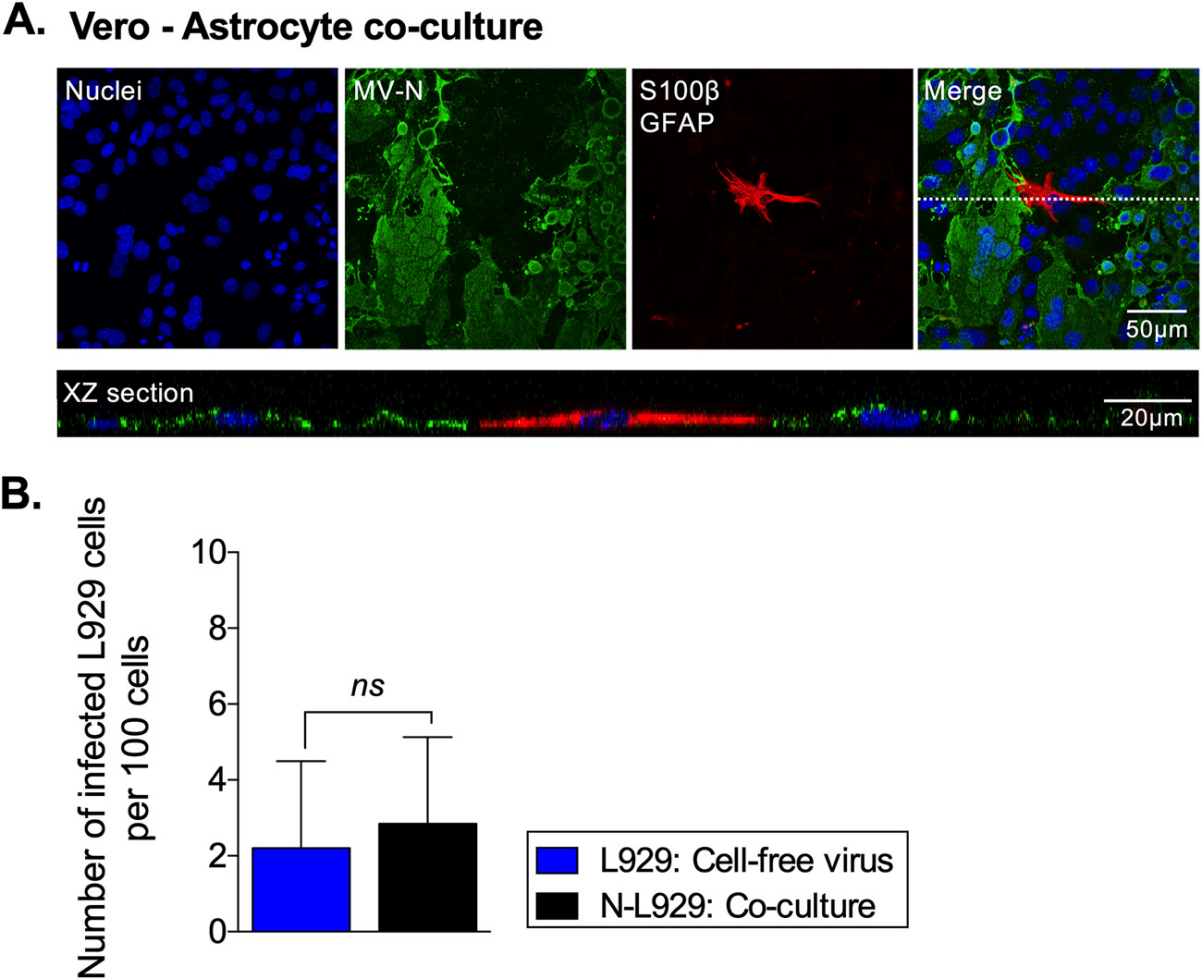
Receptor-independent MV spread from neurons to astrocytes is neuron and astrocyte specific. (A) Representative confocal images of Vero-astrocyte cocultures at 48 hpco. MV-N and the GFAP/S100β astrocyte cell markers were identified by staining with Alexa Fluor 488 (green) and 555 (red), respectively. The dotted line marks the area of the *x-z* image. (B) The number of infected L929 cells, upon inoculation with cell-free virus or upon coculture with infected neurons, was counted at 48 hpco. Data represent means with SD for three independent experiments. An unpaired *t* test for significance was used. ns, not significant.

To determine if the process of MV transmission between neurons and astrocytes is unique to these cell types, we cocultured MV-infected Vero epithelial cells with astrocytes (Vero-astrocyte) or neurons with L929 fibroblastic cells (neuron-L929). At 48 hpco, Vero-astrocyte cocultures were fixed and immunostained for viral proteins and cell markers, and images were analyzed at different focal planes by confocal microscopy. In evaluating the z-stack analysis from multiple coverslips, MV-N antigens did not colocalize with the astrocyte cell markers in any of the images, indicating that no Vero-astrocyte transfer had occurred (Fig. 4A, upper panel). The *x-z* image shows no overlay of red and green signal (Fig. 4A, lower panel). Similarly, only a small percentage of L929 cells were infected in cocultivation experiments (2.9% ± 2.3%), a value that was not statistically different from the percentage of L929 cells infected with cell-free virus (2.2% ± 2.3%) (Fig. 4B). Of note, virus loads did not increase beyond this low level, nor spread to more cells. These data demonstrate that the mode of transmission of MV from neurons to astrocytes is unique and imply that some physiological interaction between these CNS-resident cell populations is required to facilitate MV transmission.

### Astrocytes are permissive for MV reproduction and spread but do not release infectious viral progeny.

**FIG 5:**
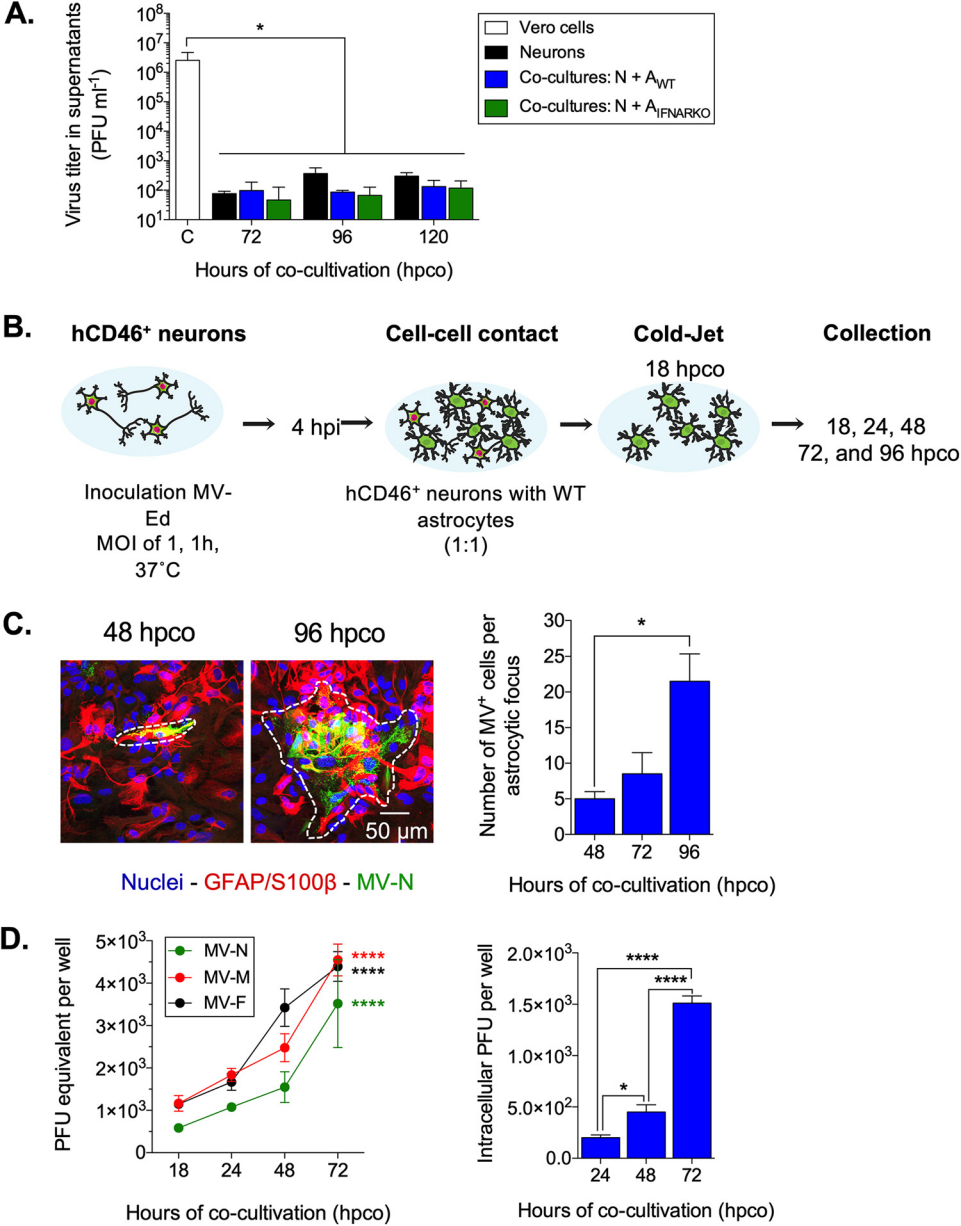
Infected astrocytes are permissive for MV reproduction and spread but do not release infectious viral progeny. (A) Extracellular virus titers from supernatants of Vero cells, neurons, or neuron-astrocyte cocultures were analyzed by plaque assay. Data shown are means with SD for three independent experiments. An ANOVA, followed by a Dunnett *post hoc* test, was used for significance; *, *P* < 0.05. (B) Schematic experimental setup. Neurons were removed selectively from the neuron-astrocyte cocultures at 18 hpco (cold jet). At the indicated time points postcocultivation, astrocytes were fixed. (C) MV-N and GFAP/S100β astrocyte cell markers were detected by immunostaining as before, with Alexa Fluor 488 (green) and Alexa Fluor 555 (red), respectively; nuclei were counterstained with Hoechst 33342 (blue). The regions marked with a white dashed line define one astrocytic focus at the indicated time points. Data represent means plus SD for three independent experiments. The number of infected cells within 30 astrocytic foci was counted. The nonparametric Kruskal-Wallis test, followed by a Dunnett *post hoc* test, was used for significance; *, *P* < 0.05. (D) RNA was collected from the remaining astrocytes at the indicated time points. (Left panel) cDNA was synthesized, followed by qRT-PCR using primers specific for MV-N, MV-M, and MV-F coding regions. Data are represented as PFU equivalent per well based on a standard curve. (Right panel) At the indicated time points, astrocytes were scraped into fresh medium, followed by a freeze-thaw cycle, to determine the intracellular virus titer using a standard plaque assay. An ANOVA, followed by a Tukey *post hoc* test, was used for significance, in which * indicates *P* < 0.05 and **** indicates *P* < 0.0001.

We next investigated if astrocytes are fully permissive for MV reproduction. Supernatants collected from the neuron-astrocyte cocultures were tested for extracellular virus particles, using plaque assay for cell-free infectious viral particles. Supernatants from infected Vero cells and neurons were included as positive and negative controls, respectively. (We have previously shown that while MV can spread within neuronal networks, no infectious progeny is released [19].) We observed no change in virus titer in the cocultures between 72 and 120 hpco, which suggests that infected astrocytes, like neurons, do not release viral progeny (Fig. 5A). Because astrocytes synthesize high concentrations of interferons in response to other neurotropic virus infections (36–38), we hypothesized that these interferons may limit the release of viral progeny from astrocytes. However, this hypothesis was not supported, as interferon alpha receptor knockout (IFNAR KO) astrocytes, which cannot respond to extracellular type I interferons (IFNs), also failed to release infectious viral particles when cocultured with neurons (Fig. 5A).

Next, neurons were selectively removed from the neuron-astrocyte cocultures at 18 hpco, resulting in >95% pure astrocyte culture (see Fig. S1 in the supplemental material; shown schematically in Fig. 5B). At 48, 72, and 96 hpi, astrocyte cold-jet cultures were fixed and immunostained for MV-N and the astrocyte cell markers. The number of infected cells within a cluster of infected astrocytes (i.e., an astrocytic “focus”) was quantified, shown in Fig. 5C. We found that MV spread efficiently between astrocytes, as the number of infected cells within an astrocytic focus increased significantly over time (*P* < 0.05), even in the absence of infected neurons. In addition, RNAs encoding different viral proteins were detected at 18 hpco in astrocytes and increased exponentially over time (*P* < 0.0001; Fig. 5D, left panel), as did the titer of virus (*P* < 0.0001 at 72 hpi; Fig. 5D, right panel). We conclude that astrocytes are permissive for viral reproduction, resulting in efficient MV spread among adjacent astrocytes. However, as with neurons, infected astrocytes do not release infectious viral progeny.

10.1128/mBio.00288-21.1 FIG S1 Neurons were removed from the neuron-astrocyte cocultures, resulting in a >95% pure astrocyte population. (A) Representative confocal images of the neuron-astrocyte coculture prior to cold-jet removal (upper panel) and the >95% pure astrocyte population, 24 h after cold jet (lower panel). The astrocyte cell markers, GFAP and S100β (left panel), and the neuronal cell marker, NeuN, are stained in red (right panel). (B) Graphical representation of the number of GFAP/S100β- and NeuN-positive cells within a confocal field of 375 × 375 μm prior to and 24 h after cold-jet removal. Twenty random fields were counted for each replicate (*n* = 3). An unpaired *t* test for significance was used for significance. **, *P* < 0.01. Download FIG S1, TIF file, 2.2 MB. Copyright © 2021 Poelaert et al. 2021 Poelaert et al. https://creativecommons.org/licenses/by/4.0/ This content is distributed under the terms of the Creative Commons Attribution 4.0 International license.

### Fusion is required for MV spread between homotypic cells of the brain but not for spread between heterotypic cells.

**FIG 6:**
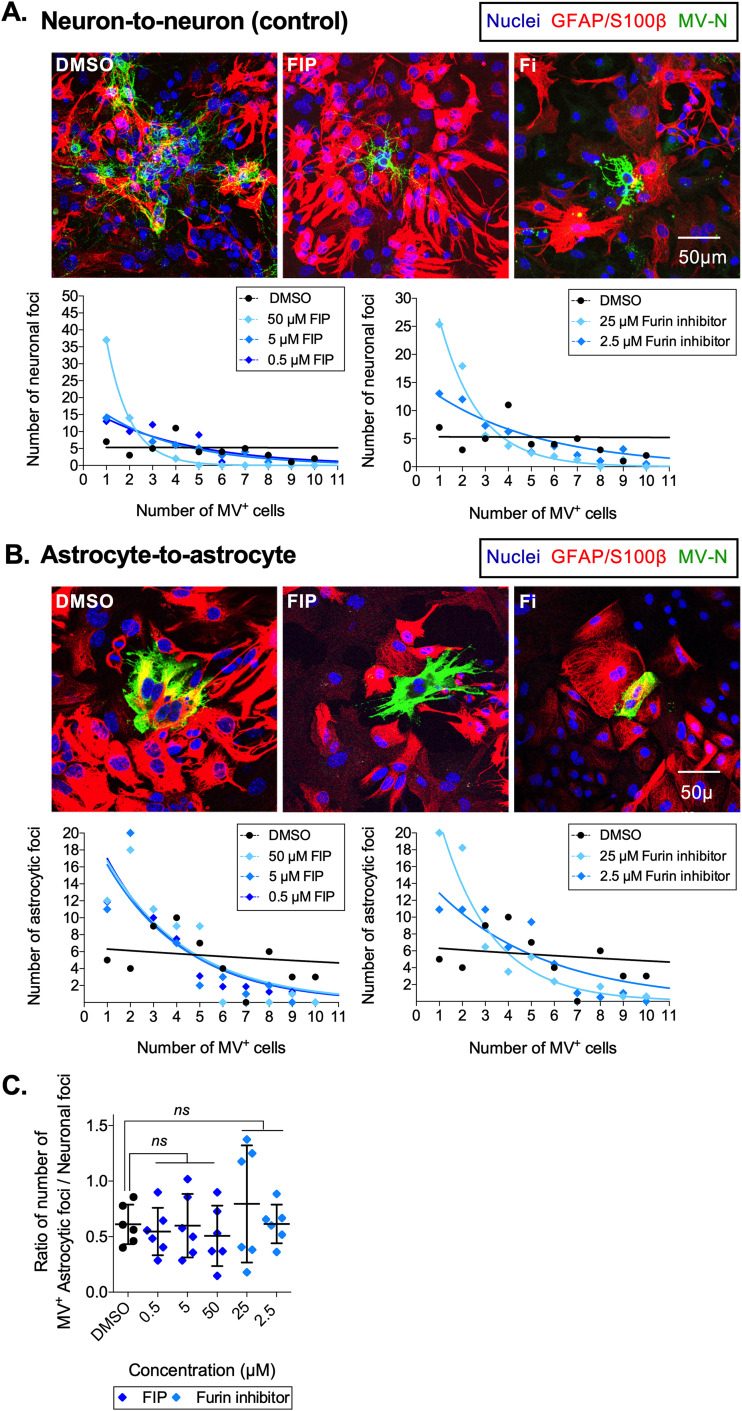
MV spread between heterotypic cell types of the brain is fusion independent. Neuronal-astrocyte cocultures were treated with two fusion-inhibitory drugs, fusion-inhibitory peptide (FIP; 0.5, 5, and 50 μM) and furin inhibitor (Fi; 2.5 and 25 μM), or equivalent volumes of DMSO. At 72 hpco, MV-N and GFAP/S100β astrocyte cell markers were identified with Alexa Fluor 488 (green) and 555 (red), respectively. (A) Representative confocal images of neuronal foci for each condition are shown (upper panel), as well as a graphical representation of the number of infected neurons within a neuronal focus for each reagent (lower panel). (B) Similarly organized data are shown for astrocytic foci for each condition. Data points for both panel A and panel B represent at least 60 foci from three independent replicates (20 per replicate). Each data point shows the cumulative total of foci containing a specific number of infected cells. The lines illustrate the trends for each reagent, plotted using GraphPad Prism. (C) The ratio of the number of astrocytic foci (numerator) over neuronal foci (denominator) within a scanning field (375 × 375 μm) was calculated. Each dot represents one replicate (*n* = 6). An ANOVA followed by a Dunnett *post hoc* test was used to gauge significance. ns, not significant.

To test the hypothesis that the mechanism of MV spread between different cell types requires fusion, we compared viral spread first within homotypic cell types (neuron-to-neuron or astrocyte-to-astrocyte) and later within heterotypic cell types (neuron-to-astrocyte) in the presence or absence of different concentrations of two fusion-inhibitory drugs, fusion-inhibitory peptide (FIP) or furin inhibitor (Fi) (17, 39–41). We included equivalent volumes of dimethyl sulfoxide (DMSO) as a negative control. For each condition, the number of infected neurons or astrocytes was counted within a focus. Figure 6A (upper panel) shows representative confocal images of neuronal foci. Note that, in both FIP and Fi images, the infected neuron is surrounded by non-GFAP-positive cells: adjacent, but uninfected, neurons. In control cultures treated with DMSO alone, an expected range of infected neurons per focus is observed (from 1 to greater than 10), shown as a horizontal trendline in Fig. 6A (lower panel). Upon treatment with FIP or Fi, however, the majority of neuronal foci contained only 1 or 2 infected neurons, shown as a downward exponential trendline in Fig. 6A (lower panel). The highest reduction in viral spread corresponded to the highest concentrations of FIP or Fi. Similar results were found in MV spread among astrocytes (Fig. 6B, lower panel).

While the above results indicate that MV induces the fusion of cell membranes in homotypic cell types (both neurons and astrocytes), results with heterotypic cell types pointed to a different mechanism. The neuron-astrocyte cocultures, treated with FIP or Fi or equivalent volumes of DMSO (negative control), are represented as the ratio of the number of astrocytic foci (numerator) over neuronal foci (denominator) within a scanning field (375 × 375 μm); 20 fields for each replicate (*n* = 6) were counted. Treatment of the neuron-astrocyte cocultures with FIP or Fi did not reduce the number of infected astrocytes (Fig. 6C), and MV transfer from infected neurons to astrocytes was not inhibited. We conclude that MV spread between neurons and astrocytes is independent of cell membrane fusion.

### EAAT glutamate transporters, but not glycine or GABA transporters, contribute to MV infection of astrocytes.

**FIG 7:**
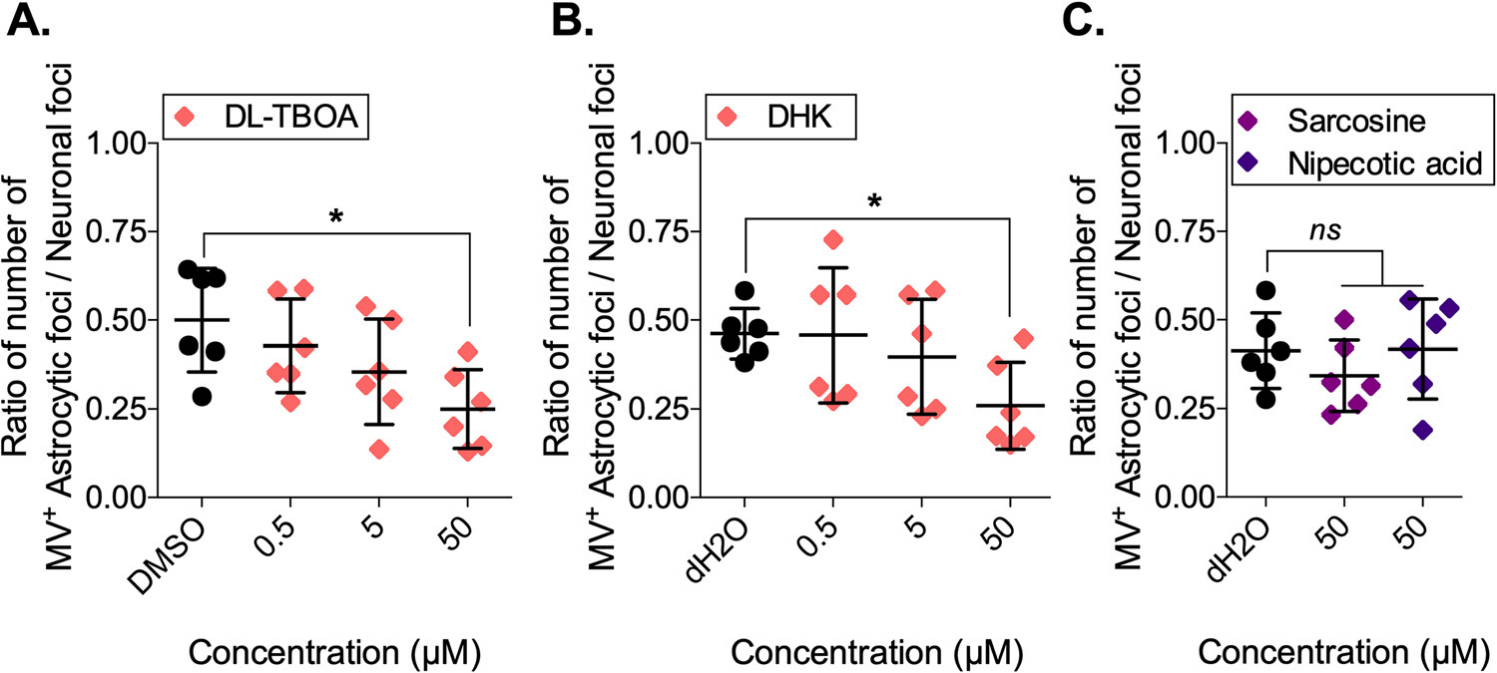
MV spread between heterotypic cell types of the CNS depends on glutamate transporters. Neuron-astrocyte cocultures were treated with dl-threo-β-benzyloxyaspartic acid (dl-TBOA; 0.5 to 50 μM), dihydrokainic acid (DHK; 0.5 to 50 μM), sarcosine (50 μM), nipecotic acid (50 μM), or equivalent volumes of DMSO or distilled water (dH_2_O) (control). At 72 hpco, neuron-astrocyte cultures were fixed, followed by IF staining and confocal analysis. The ratio of the number of astrocytic foci (numerator) over the number of neuronal foci (denominator) within a scanning field (375 × 375 μm) was calculated. (A) dl-TBOA. (B) DHK. (C) The glycine transporter inhibitor sarcosine and the GABA uptake inhibitor nipecotic acid were included as controls. Each dot represents a replicate (*n* = 6). An ANOVA, followed by a Dunnett *post hoc* test, was used for significance. *, *P < *0.05; ns, not significant.

How, then, is MV transported from neurons to astrocytes *in vitro* and *in vivo*? Considering the unique interaction between neurons and astrocytes and the importance of direct cell-cell contact to transfer mature MV, MV proteins, or genetic material, we sought to identify the astrocyte surface protein(s) that plays a role in the uptake of virus particles or viral components. Included among the potential mediators of astrocyte entry are excitatory amino acid transporters (EAAT) −1/GLAST and −2/GLT-1, which represent the majority of EAATs and are expressed mainly by astrocytes (42). We hypothesized that MV particles (complete or partial) that are released from the neuron into the synaptic cleft enter adjacent astrocytes via these EAAT receptors. To test this hypothesis, neuron-astrocyte cocultures were treated with the nontransportable EAAT inhibitors dl-threo-β-benzyloxyaspartic acid (dl-TBOA), a pan-EAAT inhibitor, and dihydrokainic acid (DHK), an EAAT-2 inhibitor. The glycine transporter inhibitor sarcosine and the gamma-aminobutyric acid (GABA) uptake inhibitor nipecotic acid were included as controls. In the presence of dl-TBOA and DHK, the number of infected astrocytic foci decreased significantly in a concentration-dependent manner (*P* < 0.05 for 50 μM) (Fig. 7A and B). In contrast, in the presence of sarcosine or nipecotic acid, no reduction in the number of infected astrocytic foci was observed (Fig. 7C). Importantly, lactate dehydrogenase (LDH) assays were performed to assess whether these compounds were cytotoxic; none of these drugs, at these concentrations, resulted in cell death (data not shown). These results implicate EAATs, but not glycine or GABA transporters, in MV transmission from neurons to astrocytes.

### RNase A treatment reduces viral spread between heterotypic cell types *in vitro*.

**FIG 8:**
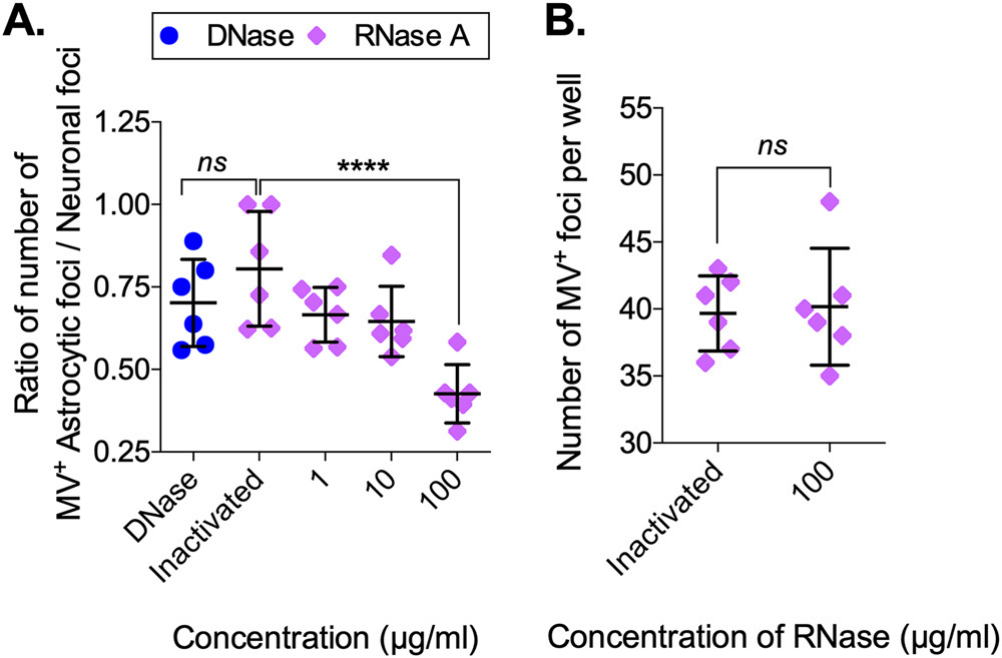
RNase A treatment reduces viral spread between heterotypic cell types. (A) Neuron-astrocyte cocultures were treated with DNase (100 μg/ml), inactivated RNase, or RNase A (1 to 100 μg/ml). At 72 hpco, neuron-astrocyte cocultures were fixed, followed by IF staining and confocal analysis. The ratio of the number of astrocytic foci over the neuronal foci within a scanning field (375 × 375 μm) was calculated. (B) Virus stock was treated with inactivated RNase or 100 μg/ml RNase A for 45 min at RT and added on top of a monolayer of Vero cells. This plaque assay was fixed at 5 dpi, and the number of viral foci was counted per well. Each dot represents a replicate (*n* = 6). An ANOVA, followed by a Dunnett *post hoc* test, was used for significance. ****, *P* < 0.0001; ns, not significant.

Finally, to determine if intact virus particles or infectious ribonucleoproteins (RNPs) are released in the synaptic cleft, neuron-astrocyte cocultures were treated with 1 to 100 μg/ml RNase A, inactivated RNase (43–45), or 100 μg/ml DNase I. The results showed a significant decrease in the ratio of infected astrocytic foci to neuronal foci in the presence of RNase A in a concentration-dependent manner (*P* < 0.0001 at the concentration of 100 μg/ml) but not upon treatment with inactivated RNase A or active DNase I (Fig. 8A). To rule out the possibility that RNase A affects RNA integrity within mature virus particles, an infectious virus stock was treated with 100 μg/ml RNase A for 1 h at room temperature (RT), followed by a plaque assay titration. No change in virus titer was observed in the presence of RNase A, compared to control (Fig. 8B). These data suggest that nonenveloped MV RNA, perhaps in the form of ribonucleoproteins, is secreted into the synaptic cleft, followed by uptake into astrocytes.

## DISCUSSION

Viruses are the most common cause of encephalitis in mammals worldwide. Although the morbidity of viral encephalitis is low (∼7.4 per 100,000 persons per year), persistent clinical challenges and mortality are high in untreated patients (70 to 80%) (46). Numerous viruses are associated with encephalitis, including herpes-, flavi-, entero-, and paramyxoviruses. Here, we used measles virus (MV) and hCD46^+^-NSE transgenic mice as a model to evaluate viral tropism within the brain and to assess how this virus spreads among distinct parenchymal cell populations. In this model, the MV receptor hCD46 is expressed only by neurons, and consequently, only these cells can become infected directly with MV. Although these mice differ in fundamental ways from the human CNS, we contend that such studies allow us to explore the dependence of receptor expression on viral transmission among different cell types within the brain.

Our *in vivo* experiments demonstrate that the majority (>90%) of the infected cells in NSE-hCD46/RAG KO mice at 30 dpi express the neuronal cell marker NeuN; however, a small proportion (5 to 10%) costained with the astrocyte cell markers GFAP and S100β. Interestingly, upon immunosuppression at 90 dpi, we observed a shift from neurons to astrocytes as the predominant infected cell type (>80%). This suggests that once astrocytes are infected, they become the main cell type in which the virus is dormant. Whether MV is cleared from infected neurons, or if these neurons eventually die, remains a question of interest. Nevertheless, the observation that astrocytes serve as a shelter for MV during latency or persistence is consistent with studies for other viruses, including mouse hepatitis virus (47).

In this paper, we refer to dormancy, which we suggest is a viral state functionally distinct from both latency and persistence. Latency is a defining feature of herpesviruses, in which the virus is present in the absence of viral transcription and translation. In persistent infections, such as with Ebola virus, the virus continues to reproduce and release infectious progeny from infected cells (48, 49). Recently, we introduced the term “dormancy” for the chronic nontransmissible reproductive state of MV within the CNS (16). In this scenario, MV remains in infected CNS cells, with transcription and genome replication occurring at low levels, but in the absence of progeny production and attendant disease. Viral replication can recur at some point long after challenge, with production of viral progeny and renewed spread. In this study, we show that astrocytes are the predominant cell population that maintains dormant MV within the CNS.

Primary astrocytes isolated from hCD46^+^-NSE mice lack hCD46 and are therefore not susceptible to infection by purified measles virus particles. Astrocytes become infected only upon direct cell-cell contact with infected neurons. Once in the astrocyte host, MV can reproduce efficiently and spread from one astrocyte to another without the release of infectious viral progeny, resulting in formation of astrocytic foci. Unlike infection of fibroblasts or epithelial cells, syncytia were not observed by confocal microscopy. Previously, S. Schneider-Schaulies et al. (50) demonstrated restricted MV gene expression of the envelope proteins M, F, and H in rat astroglial cultures, indicating an abortive MV infection. In contrast, we found that MV genes and proteins are expressed in the murine astrocytes and spread over time in culture, suggesting productive viral replication in the absence of progeny production (Fig. 5). The inability of cell-free MV to infect astrocytes directly and the efficiency of viral spread among astrocytes without the release of viral progeny make this a particularly nimble model to study mechanisms of viral spread within (e.g., neuron-neuron or astrocyte-astrocyte) and among (neuron-astrocyte) parenchymal cell populations.

Using this mouse model, and primary cells obtained from these mice, we confirmed that MV spread among homotypic cell types of the brain occurs by fusion of the cell membranes of the donor and recipient cell. This result is consistent with that described for interneuronal MV spread in previous studies (19, 51–54). Both interneuronal and interastrocytic MV spread are hCD46 independent, which implicates either the presence of an unknown, but perhaps related, MV receptor or some mechanism that does not rely on a cellular receptor. It is not surprising that viral spread between homotypic cell types occurs similarly, as it is known that homotypic adhesion molecules allow viruses to spread from an infected donor to an adjacent homotypic recipient cell (55).

The study of how MV spreads within a cell population has been a main focus for many researchers. However, prior to our investigation, the mode of viral transmission between heterotypic cell types (here, neuron to astrocyte) had not received much attention but may be quite relevant *in vivo*. A recent study revealed the importance of the interaction of nectin-1 and -4 in the heterotypic MV spread in the respiratory tract between epithelial and neuronal cells (13). Our results using cells cultured directly *ex vivo* indicate that MV transmission from hippocampal neurons to astrocytes is unique to these cell populations: swapping out other permissive cells did not result in efficient viral transmission, implying that neuron- and/or astrocyte-specific surface proteins and/or machinery is critical for MV transmission. To characterize this cellular relationship more deeply, we employed specific compounds, each with a unique mode of action. We found that, in contrast to homotypic cell spread, viral spread between neurons and astrocytes occurs without fusion of the (synaptic) membranes. We hypothesized that MV is released into the synaptic cleft and interacts with specific astrocytic proteins and signaling pathways associated with neurotransmitter reuptake. Maintenance of homeostasis of synaptic neurotransmitters, including glutamate, GABA, and glycine, is a key function of astrocytes at the tripartite synapse (42, 56). As neurotransmitter transporters are arrayed on the astrocyte cell surface (56–58), we hypothesized that viral particles or proteins might be taken up by neurotransmitter reporters. Although blocking glycine or GABA transporters did not prevent astrocyte infection, selective inhibitors of the glutamate transporters (EAATs) reduced astrocyte infection significantly. Both dl-TBOA and DHK target EAAT-1, which is predominantly expressed by CNS astrocytes throughout the brain, consistent with the distribution of MV during dormancy (15, 59). These results suggest that MV spreads from neurons to astrocytes via an essential physiological pathway of the CNS. Remarkably, MV is not the only virus interacting with one of the latter neurotransmitter transporters; both human T-cell lymphotropic virus type 1 (HTLV-1) and bovine and murine leukemia virus (BoLV and MuLV) bind to this family of transporters to enable entry into a cell (60–63); thus, this may be a more generalized way by which neurotropic viruses are disseminated within the CNS.

To initiate studies to determine the nature of the “infectious unit,” we treated cocultures with RNase, reasoning that nonenveloped RNAs would be degraded but that complete particles would not. The significant reduction in the number of infected astrocytes when treated with RNase suggests that nonenveloped RNA, perhaps in the form of ribonucleoproteins, is released into the synaptic cleft. While this hypothesis requires further testing, it is consistent with the lack of dependence on either canonical receptors or the viral fusion machinery. Infectious RNA has been described for other RNA viruses, including dengue virus (64) and Sindbis virus (65). Interestingly, Singh et al. (66) showed a rapid lateral spread of RNPs among human respiratory epithelial cells. This type of atypical viral spread likely protects the virus from the hostile extracellular environment and may evade detection and neutralization by antibodies. Such a “safe” microenvironment is presumably also created in the tripartite synaptic complexes in which astrocytic end feet surround the pre-post neuronal synaptic junction.

In summary, our studies indicate that viral spread within the brain may be exquisitely sensitive to characteristics of both the donor and recipient cell type. We propose that MV, in some form, is released into the cleft, where it can then engage with glutamate transporters, either directly using these proteins as a receptor or indirectly as a coreceptor. Ultimately, infection of astrocytes is established and—in the case of MV—remains the site for long-term dormancy. In periods of immune suppression, viral RNA and protein synthesis rebound in astrocytes, resulting in viral transmission to neurons and reinitiating neuropathogenesis, months or even years after initial exposure. This novel mode of neurotropic viral transmission paves the way for a follow-up study to address how the infectious RNP is released into the synaptic cleft and how interaction with neurotransmitter transporters aids entry. We believe such studies enrich and broaden our understanding of viral transmission, especially in organs with closely interacting, heterogenous cell populations, and reveal potential therapeutic targets to preclude or ameliorate potentially catastrophic effects when viruses invade the brain.

## MATERIALS AND METHODS

### Ethical approval statement.

Mice were maintained in the Association for Assessment and Accreditation of Laboratory Animal Care (AAALAC)-accredited laboratory animal facility at the Fox Chase Cancer Center. This study was carried out in the accordance with the recommendations provided in the *Guide for the Care and Use of Laboratory Animals* (68). The experiments were conducted in compliance with the protocols approved by Fox Chase Cancer Center Institutional Animal Care and Use Committee (Office of Laboratory Animal Welfare assurance number A3285-01).

### Virus and cell lines.

The MV-Edmonston (MV-Ed) strain (VR-24; ATCC, Manassas, VA) was propagated and quantified on monolayers of Vero cells (CCL-81; ATCC) according to standard protocols. Vero cells and L929 murine fibroblasts (NCTC clone 929 CCL-1; ATCC) were maintained in complete Dulbecco’s modified Eagle’s medium (DMEM; Gibco, Invitrogen, Carlsbad, CA), containing 10% fetal bovine serum (FBS; HyClone Laboratories, Inc., Logan, UT), 100 U/ml penicillin (Gibco), 100 ng/ml streptomycin (Gibco), and 2 mM l-glutamine (Corning Life Sciences, Corning, NY).

### Experimental models. (i) *In vivo* infection model.

*(a) Mice.* Homozygous NSE-hCD46^+^ transgenic mice (line 18; H-2^b^) (15), RAG2 KO/NSE-hCD46^+^ mice, and interferon alpha receptor knockout (IFNAR KO) mice were maintained in the closed breeding colony of the Fox Chase Cancer Center and within the biosafety level 2 facility. The genotypes of mice were confirmed by PCR analysis of tail biopsy specimen DNA and/or blood samples collected from the retro-orbital sinus. In this study, males and females between 6 and 8 weeks of age at the beginning of each experiment were used. No differences in viral loads or pathogenesis have been observed between adult males and females.

*(b) Infections.* Mice were anesthetized with isoflurane and injected intracranially along the midline with 30 μl inoculum, containing 1 × 10^4^ PFU MV-Ed, using a 27-gauge needle. The infected mice were monitored daily for disease symptoms (weight loss, ruffled fur, ataxia, and seizures). Moribund mice were euthanized in accordance with IACUC guidelines. At indicated time points, isoflurane-anesthetized mice were injected intraperitoneally with 0.5 ml of 3.8% chlorohydrate. Mice were perfused intravascularly with 4% paraformaldehyde (PFA; Electron Microscopy Sciences, Hatfield, PA) diluted in phosphate-buffered saline (PBS), to preserve the brain for subsequent sectioning and immunohistochemistry. Brains were snap-frozen in tissue embedding compound (Tissue-Tek O.C.T. compound; Sakura, Torrance, CA) using dry ice and isopentane. Axial (horizontal) brain sections of 10 μm were made and fixed in 2% PFA containing 2% sucrose (15 min, RT) and in 95% ethanol (20 min, −20°C) and permeabilized with 0.2% Triton X-100 (30 min, RT).

(*c*) *Irradiation and bone marrow chimera generation.* Irradiation and bone marrow chimera generation were carried out as previously described (16). Briefly, MV-infected mice (±90 days postinoculation [dpi]) were subjected to lethal panoramic gamma irradiation (2 doses of 5.5 Gy separated by 4 h) using a Shepherd model 81-14R Cs-137 panoramic irradiator. Concurrently, bone marrow was extracted from the femur and tibia of multiple, uninfected RAG2 KO donor mice and pooled. Red blood cells were depleted using ACK lysis buffer (Gibco), and the remaining cells were washed twice with sterile PBS. A total of 1 × 10^7^ cells was diluted in 150 μl PBS and administered to irradiated recipient mice via the retro-orbital sinus, 18 h after the final irradiation of the recipients.

### (ii) Isolation of primary hippocampal neurons and cortical astrocytes.

*(a) Primary hippocampal neurons.* Primary neurons were prepared from murine NSE-hCD46^+^ embryonic hippocampi (E15 to −16), as described previously (69–72). Neurons were plated on 15- or 18-mm glass coverslips coated with poly-l-lysine (PLL; Sigma-Aldrich, St. Louis, MO) or directly in PLL-coated 12-well plates, at a density of 2 × 10^5^ cells per well in complete DMEM. At 4 h postplating, the medium was replaced by neurobasal medium (Gibco) containing 2% B-27 supplement (Gibco), 1% l-glutamine, 1% penicillin-streptomycin, and 500 μl glutamic acid (25 mM; Sigma-Aldrich). Neurons were incubated for 2 days to allow for full differentiation prior to MV inoculation.

*(b) Primary cortical astrocytes.* Primary astrocytes were isolated from murine NSE-hCD46^+^ embryonic hemispheres (E15 to 16), using a protocol adapted from the work of S. Schildge et al. (73). Briefly, the meninges, choroid plexus, and hippocampus were removed, and the cortices were placed in 0.25% trypsin for 30 min at 37°C. The tissues were dissociated and pipetted using a heat-polished Pasteur pipette, and the cell suspension was transferred into a 70-μm cell strainer. Astrocytes isolated from 4 hemispheres were pooled and cultured in a PLL-coated T75 cell culture flask (Corning Life Sciences) in complete DMEM. After 3 days postisolation, the medium was replaced with fresh complete DMEM. Once confluent, astrocytes were detached with 0.04% trypsin, seeded into a PLL-coated T175 cell culture flask, and grown until confluent. Astrocytes were inoculated or cocultivated with neurons at the 2nd to 5th passage.

### (iii) Infection of primary hippocampal neurons and cortical astrocytes.

Primary neurons and astrocytes were exposed to MV for 1 h at 37°C at a multiplicity of infection (MOI) of 1, based on the titer of infectious virus particles obtained in Vero cells. After 1 h, the virus inoculum was removed, cells were washed three times with prewarmed PBS to remove residual unbound particles, and 2 ml of complete neurobasal medium was added to each well. At indicated time points postchallenge, cells were fixed with 4% PFA-4% sucrose in PBS (15 min, RT) and 100% ice-cold methanol (10 min, RT), followed by permeabilization with 0.2% Triton X-100 (10 min, RT). Alternatively, cells were washed with PBS and collected in 350 μl RLT buffer (Qiagen GmbH, Hilden, Germany) supplemented with 1% β-mercaptoethanol, for RNA isolation.

### (iv) Cocultivation assays.

Primary astrocytes were washed with PBS and detached from the cell culture flask with 0.04% trypsin. Astrocytes were resuspended in complete neurobasal medium, and cells were quantified using a Countess cell counter.

*(a) Indirect cocultivation.* Astrocytes were plated on 15-mm glass coverslips coated with PLL at a density of 2 × 10^5^ cells per well in complete DMEM. At 24 h postplating, the medium was removed from the astrocytes and replaced with complete neurobasal medium. Inoculated neurons (4 hpi), seeded onto 15-mm coverslips, were then inverted over astrocytes. The astrocytes were separated from the neurons by a small bead of paraffin wax (approximately 5 mm), as previously described (33), and cocultured for 72 h.

*(b) Direct cocultivation.* Wild-type (WT) or IFNAR KO astrocytes, isolated from NSE-hCD46^+^ or IFNAR KO mice, respectively, or L929 fibroblasts (control) were directly added to wells containing MV-inoculated neurons or Vero cells (4 hpi) at a ratio of 1:1. The neuron-astrocyte cocultures were incubated at 37°C during 24, 48, 72, or 96 h postcocultivation (hpco). The Vero-astrocyte (V-A) and neuron-L929 (N-L929) cocultures were incubated during 48 hpco. If indicated, fusion-inhibitory peptide (FIP; *Z*-d-Phe-l-Phe-Gly-OH; Bachem, Bubendorf, Switzerland), Furin I inhibitor (Sigma-Aldrich), *N*-methyl-d-aspartate (NMDA; Sigma-Aldrich), dl-threo-β-benzyloxyaspartic acid (dl-TBOA; Tocris), dihydrokainic acid (DHK; Tocris), nipecotic acid (Sigma-Aldrich), sarcosine (Sigma-Aldrich), DNase I (Sigma-Aldrich), or RNase A (ThermoFisher Scientific) was added to the neuron cultures, at the same time that the astrocytes were seeded (0 hpco) and maintained in the cultures throughout the experiment. RNase A was inactivated using UV light for 1 h at RT (45). At indicated time points, cocultures were fixed with 4% PFA-4% sucrose in PBS (15 min, RT) and 100% ice-cold methanol (10 min, RT) and permeabilized with 0.2% Triton X-100 (10 min, RT).

### (v) Cold-jet neuron removal.

When indicated, neurons were removed from the direct neuron-astrocyte cocultures as described previously (74). Briefly, at 18 h postcocultivation (hpco), the 12-well tissue culture plates were placed on ice and the medium was removed gently from the well. The cells were rinsed with ice-cold PBS, and PBS was pipetted three times directly onto the cells using concentric circular motions, ensuring the force of the stream contacted the entire surface of the well. This resulted in the detachment of neurons from the cocultures, leaving a >95% pure astrocyte culture in the well. Astrocytes were collected at 18, 24, 48, and 72 hpco in RLT buffer for RNA isolation. In parallel, astrocytes cultured on coverslips were fixed at 48, 72, and 96 hpco in 4% PFA-4% sucrose, followed by 100% methanol as described above. At the indicated time points, astrocytes were washed with PBS, and 500 μl complete DMEM was added per well. Cells were scraped into the medium, followed by a freeze-thaw cycle and a plaque assay to determine virus titer.

### Quantitative reverse transcriptase real-time PCR (qRT-PCR).

RNA was purified from Vero cells, primary neurons, and astrocytes using the RNeasy minikit (Qiagen, Hilden, Germany) with a final elution volume of 30 μl, following the manufacturer’s recommendations. RNA was quantified using a Nanodrop spectrophotometer and reverse transcribed using a high-capacity cDNA reverse transcription kit (Applied Biosystems) with random hexamer priming. Gene-specific primer pairs were designed using the antigenome of MV. The primer pairs were used in combination with probes designed using the Universal Probe Library system (Roche) and Universal Master Mix (Roche). All reactions were run on an Applied Biosystems QuantStudio 6 Flex machine and analyzed using the QuantStudio software. Cycling conditions were 50°C for 2 min and 95°C for 10 min, followed by 40 (2-step) cycles of 95°C for 15 s and 60°C for 60 s. Relative quantification to the control (cyclophilin B) was done using the comparative threshold cycle (ΔΔ*C_T_*) method. A standard curve of RNA from a known amount of PFU was run to determine PFU equivalents. Individual sample PCRs were performed in triplicate. Gene-specific primers (Integrated DNA Technologies, Coralville, IA) used in this study are listed in Table 1.

**TABLE 1:** Gene-specific primers used for qRT-PCR in this study

Primer	UPL^*a*^	Sequences (5′ to 3′)	
Cyclophilin B	20	Forward	TTC TTC ATA ACC ACA GTC AAG ACC
		Reverse	ACC TTC CGT ACC ACA TCC AT
MV nucleoprotein	80	Forward	GGA AAC TGC ACC CTA CAT GG
		Reverse	GGG TAT GAT CCT GCA CTG AAC T
MV hemagglutinin	5	Forward	AAA TTG GAT TAT GAT CAA TAC TGT GC
		Reverse	CAG TAG AGT TGA GTT CAC CAA TG
MV matrix protein	62	Forward	AAT TCA GAT CGG TCA ATG CAG
		Reverse	CCT ATC GCC TTG TCA ATC CT
MV fusion protein	88	Forward	GAC AAA TCT GGG GAA TGC AA
		Reverse	GGT CCG ATG ACT CCA ACA AT

a UPL, Universal Probe Library (Roche).

### Immunofluorescence staining of brain sections and cell cultures.

Brain sections and cell (co-)cultures were rinsed three times in PBS and treated with 10% normal goat serum (Vector Laboratories Inc., Burlingame, CA) and 5% FBS diluted in PBS (45 min, RT). The nonspecific IgG binding sites (e.g., murine leukocytes) in the brain sections were blocked with 0.1 mg/ml AffiniPure Fab fragment goat anti-mouse IgG (H+L) (Jackson ImmunoResearch, West Grove, PA) diluted in PBS (60 min, RT). MV nucleoproteins (MV-N) were detected in brain sections and cell cultures using the monoclonal mouse anti-MV-N-antibody (1:3,000; MV-N hybridoma clone 25; Sigma-Aldrich; P. Giraudon and T. F. Wild [75]) diluted in PBS (60 min, RT), followed by incubation with directly conjugated Alexa Fluor 488 secondary antibodies (1:5,000; Life Technologies). Neurons and astrocytes were labeled with polyclonal rabbit anti-NeuN antibody (1:2,500; Abcam) or polyclonal rabbit anti-GFAP (1:2,500; Novus Biologicals, Centennial, CO) and anti-S100β (1:2,500; Novus Biologicals) antibodies, respectively, followed by incubation with directly conjugated Alexa Fluor 555 secondary antibodies (1:5,000; Life Technologies). Nuclei were counterstained with Hoechst 33342 (1:750; ThermoFisher Scientific, Waltham, MA) diluted in PBS. Sections and cell cultures were mounted in CitiFluor AF1 (Electron Microscopy Sciences) and sealed. Images were captured at a magnification of ×40 using an inverted Leica SP8 confocal microscope.

### Plaque assay. (i) Virus titration.

Vero cells were seeded in 6-well plates at a density of 1 × 10^6^ cells per well. The next day, 10-fold serial dilutions in complete DMEM were made of the supernatants derived from inoculated Vero cells, primary neurons, direct neuron-astrocyte cocultures, and the cold-jet experiment. Vero cells were rinsed in PBS and incubated with the diluted samples for 60 min at 37°C. After 1 h of incubation, the monolayer was overlaid with 4 ml of 0.5% agarose (Sigma-Aldrich) diluted in complete DMEM. After incubation at 37°C for 5 days, the cells were fixed with formaldehyde, diluted in PBS (1:3) for 30 min. The agarose overlay was discarded, and the plaques were visualized by staining the monolayer with 0.1% crystal violet in 20% ethanol.

### (ii) RNase pretreatment.

A serial dilution of the virus stock was made. The dilution of 10^−6^ was pretreated with 0 or 100 μg/ml RNase A for 40 min at RT. Next, Vero cells were incubated with the samples for 60 min at 37°C and overlaid with agarose as previously described.

### Statistical analysis.

Data representation and statistical analysis were performed using GraphPad Prism 6 software. Statistical analysis was performed by one-way analysis of variance (ANOVA) followed by a multiple-comparison test unless otherwise specified. A value of *P* < 0.05 was considered statistically significant.
