# Supplementary figures and images for: Noncanonical Transmission of a Measles Virus Vaccine Strain from Neurons to Astrocytes
Source: mBio. 2021 Mar 23;12(2):e00288-21. doi: 10.1128/mBio.00288-21 (PMC8092232; doi:10.1128/mBio.00288-21)

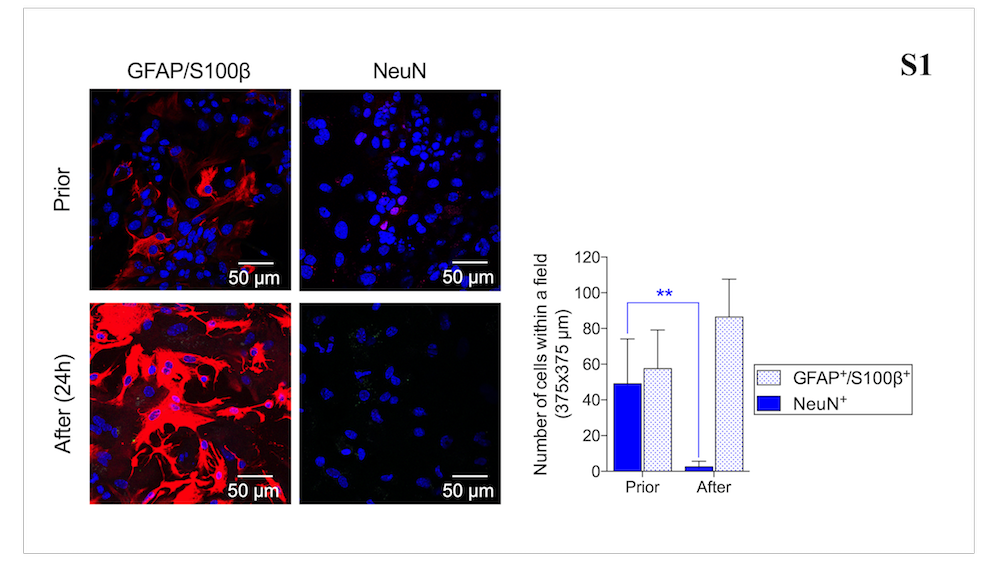

Supplement: FIG S1 [file mBio.00288-21-sf001.tif]
